# Perceived Body Appearance and Eating Habits: The Voice of Young and Adult Students Attending Higher Education

**DOI:** 10.3390/ijerph16030451

**Published:** 2019-02-04

**Authors:** Tali Heiman, Dorit Olenik-Shemesh

**Affiliations:** Department of Education and Psychology, The Open University of Israel, Raanana 4353701, Israel; doritol@openu.ac.il

**Keywords:** body-image perception, adults, appearance, habits

## Abstract

This study examined the relationship between social-environmental influences and body image perception. Specifically, the study explored the perceived body appearance among young and older students attending higher education, and their eating experiences, as related to four main social-environmental circles: family, friends, work colleagues, and media. The present study interviewed 30 students (14 men and 16 women) ages 20–40. The findings show that most of the participants were concerned about their appearance, reported on various eating habits rooted on family tradition. Findings revealed differences among gender and age groups regarding, especially regarding sport and dissatisfaction with their own perceived appearance. Although more women reported on healthy eating habits and doing sport, they reported higher dissatisfaction with their body appearance. It was found that in both groups, family habits and their parents’ remarks about bodies had an ongoing effect and significant influence on their body self-perceptions. Further health promotion should be directed in particular to individuals with a perceived negative body image, in order to enhance positive body self-perception, implementing heathy eating habits and engaging higher commitment to sport.

## 1. Introduction

### 1.1. Perceived Body Appearance 

Body image is defined as an individual’s thoughts, feelings, and behaviors associated with his or her appearance and physical ability [1]. The interest in body appearance has been both theoretical and practical, in various fields of the social sciences [2] including examining the relationships between perceived body image and different psychological and health issues such as depression, eating disorders, and low self-esteem [3,4]. Only a few studies have examined body image while focusing on social environments among the adult population, therefore the present study focused on the relationship between body perception, gender, and age, through an in-depth exploration of how adults disclose their appearance and how much the surroundings (i.e., family, peers, media) influence on their body perception. In addition, this study intends to examine variables inherently related to body image, such as eating and exercise habits, media exposure, and so on. 

### 1.2. Gender, Age, and Perceived Body Appearance 

Gender differences related to body appearance are a well-known and well-documented phenomenon [4,5,6]. Gender differences emerge in childhood and seem to persist across most of one’s lifespan, and are usually attributed to Western culture, where the media and socialization agents tend to illustrate female beauty ideals [7]. These beauty representations usually set extremely high practically unachievable standards. Literature reviews indicated that body appearance issues and eating disorders are more prevalent in women, especially adolescents and young adults [8,9]. Especially notable is the emphasis on thinness in women across all age groups, and the overall presentation of the female body as attractive rather than functional. The cultural construction of the ideal body for men versus women has various emotional, cognitive, and behavioral consequences. 

International comparative studies conducted by the World Health Organization (WHO) in 1994 (including 28 Western countries) and during 1998 (including 44 Western countries) concluded that the frequency of dieting behaviors among Jewish-Israeli adolescents was the highest of all participating countries [10]. Findings of an Israeli youth sample (ages 13–18) revealed that 47.1% of participants defined their own body shape as full, fat, or very fat; 45.3% expressed a wish to lose weight. Moreover, 75.8% of participants who described themselves as having a full body shape and 36.4% of those who described themselves as fat were within the normal range, based on their Body Mass Index (BMI) scores [11]. Compared with other countries, Israeli adolescents emerge as being exceptionally dissatisfied with their bodies. The study revealed that the frequency of maladaptive eating habits among both male and female Jewish adolescents is high compared to most other Westernized countries. For example, 60–80% of female Israeli adolescents are dissatisfied with their weight and shape, although a majority of these adolescents are of normal weight. In Israel, as well as in other Western countries, women in particular appear to be less satisfied with their body’s appearance and become more vulnerable to low body image [12,13]. 

Age seems to be another important factor when studying body image. As mentioned before, most of the research on body image has focused on younger populations as children, adolescents, and young adults, whereas studies focusing on older adults remain limited. Available data suggest that older adults experience sociocultural pressures to conform to youthful appearance ideals pressures that affect one’s perceived body image [14]. On the other hand, it was found [15,16] that with age, some people become less interested in their appearance and are less prone to conform to society’s expectations, suggesting age might act as a predictor of positive body image. 

Purpose of this study was to explore body appearance perception among adults, and to examine how it is related to four main social-environmental circles: family, friends, peers, and the media.

### 1.3. Family Circle

Studies on the effects of family characteristics and body image are mostly concerned with eating disorders [8]. Parents’ actions and beliefs, their implicit and explicit communication with the child, have a significant effect on the child [17,18,19]. Parents’ messages regarding the child’s body (shape, size, weight, etc.), or encouragement the child to lose/gain weight or to eat health food, had a strong predictor of the child body dissatisfaction [20,21]. Generally speaking, Israeli society (both Jewish and Arab) emphasizes family relationships and tends to keep close familial ties as compared to other nations. Katz [11] showed that the value of “spending time with family” was one of the most important issues for Israelis and placed at the top of a list comprising 35 values. In Harel-Fish et al.’s survey, approximately 91% of Israeli participants reported they could easily speak with their parents (76% spoke mostly with their father, and 88% spoke mostly with their mother); this finding placed Israel in eighth place out of 42 countries. Most of the Israeli participants reported that their parents knew what was going on in their children’s lives [10].

### 1.4. Friends and Peers Circle

In a rapidly changing society, peer groups have a great influence on an individual. Therefore, in addition to family and the media, one’s peer group and friends strongly influence the process of socialization and become ‘significant others’. Nowadays, social comparisons are related to the development of body dissatisfaction among children, adolescents, and adults. Tatangelo and Ricciardelli [22], in examining the impact of peers and media on body-image perception among children, found that appearance comparisons are more common among women, whereas sports/ability comparisons are more common among men. In addition, men viewed media comparisons as inspiring, whereas women reported negative emotions in response to such comparisons. Studies conducted on college students, examining the relationships between perceived body image, appearance, and the effects of peers [23] revealed that peer pressure is unavoidable and most often negative, especially for women. Yet, intensified parental control can also negatively influence on how college women and men feel about their bodies. Another study [24] examining the relationship between peer influence, media exposure, and body-image satisfaction in young adults showed that media exposure was higher among females than males. 

### 1.5. Media Circle

Several studies have found significant correlation between exposure to Western media and body dissatisfaction [4,25,26]. Other studies have found a decrease in body satisfaction following exposure to media images, typically in women presented with the “thin ideal” [27,28]. An examination of self-perception among women on Facebook revealed that women high in appearance comparison reported more body dissatisfaction after spending time on Facebook, compared to a control site group [29,30]. Studies on the effects of exposure to video games on body image in men and women yielded similar results [31,32].

International studies conducted by the WHO between 1994 and 2014 mapped Israeli adolescents’ media exposure and usage. As Harel-Fish et al. [10] reported, the survey shows that Israeli adolescents are more exposed to media than most of their peers in other countries. A cross-culture study [33,34] including a sample of young and middle-aged adults examined body-weight satisfaction, among a Mediterranean adult population, show that female participants were more concerned about their body-weight status, more dissatisfied being overweight, and more worried about weight gain than men. Moreover, middle-aged participants were more dissatisfied with their body shape and underestimated their body weight more than the younger adults. 

The present study intended to deepen our understanding of the influence of the individual’s social environment, in particular its influence on the individual’s habits, behaviors, and body appearance perception. It was expected that the main differences would emerge for gender and for age group. As such, women would be more concerned about their appearance and less satisfied with their body, and that their appearance self-perceptions would be lower than those of the men. 

## 2. Materials and Methods

### 2.1. Measures

A semi-structured interview was created based on the European Cooperation of Scientific and Technical Research Program entitled: “Appearance Matters: Tackling the psychical and psychosocial consequences of dissatisfaction with appearance”. 

The interview comprised three topics: (a) three questions referring to body awareness and body image, including personal perception of body awareness and body image, and habits concerning the body, such as, “What do you think about your body?” and “Are you doing any sports?” (b) four questions regarding the impact of agents of socialization on their body image (e.g., family members, media, friends, colleagues), such as “Did your family members express any judgments, thoughts, and feelings about your body and body appearance?” and “Did your friends, peers, and colleagues express any judgments, thoughts, and feelings about your body and body appearance?” and (c) two questions regarding the impact of perceived body image and appearance on workers’ career, such as “Do you think that your body image and appearance have had influence in your working career?” and “Do you think that your body image and appearance have affected your career promotion?” (see Appendix A)

In addition, personal background was recorded (gender, age, education, marital status). 

### 2.2. Participants 

Thirty (14 men, 16 women) adult students, born in Israel, agreed to be interviewed as participants for this study. They were recruited through researchers’ professional networks, through "snowball" sampling. All of the participants were learning at the higher education institutes in Israel, located in large cities and in the suburbs, at the center of Israel. The participants present a middle and upper socioeconomic class and belongs to Israeli-Jewish culture. 

They were ages 20–40 (mean age, men = 28.5, S.D = 4.7; mean age, women = 26.87, S.D = 5.46). A chi-square analysis revealed no significant differences between gender and age, χ^2^ (1, *N* = 30) = 12.94, *p* = 0.23. However, due to the large age range, the participants were divided into two age groups based on the mean age of the entire participant group: 20–30 years and 31–40 (mean age: young men = 24.87, S.D = 1.55; older men = 33.3, S.D = 2.25; young women = 23.2, S.D = 0.92; older women = 33.17, S.D = 3.43). In order to examine differences in between each of the age groups and gender, two chi-square analyses were conducted. No significant differences were found between age and gender in the younger group, χ2 (4, *N* = 18) = 6.48, *p* = 0.16, and not for the older group, χ^2^ (5, *N* = 12) = 6.00, *p* = 0.31. As Table 1 shows, the participants’ backgrounds included a similar distribution of gender, marital status (unmarried, married, divorce), education (bachelor’s degree (BA), and master’s degree (MA), and age. All the students studied social science, or a mixture of humanities and social sciences. 

### 2.3. Procedure

After receiving the ethical approval of The Open University (ethical approval # 2988) the research team interviewed the participants. The research team consisted of the two authors and a master’s degree student assistant who was familiar with the subject, specialized in interviewing and in conducting qualitative analysis. 

The potential benefits of a qualitative approach include the opportunity to access meanings, perspectives, and interpretations from different groups of people [35]. Therefore, by conducting the semi-structured interview, we were able to interview each participant face to face or by telephone for about 60–90 minutes. All the interviewees agreed to be recorded, and all identifiable information was removed to ensure confidentiality. 

Data Analyses: Qualitative analysis was conducted to identify themes in the data. The authors conducted thematic analysis manually, because no previous studies had examined the relationship between body appearance and the ecological aspects of social-environmental circles. The authors and the research assistant separately coded the themes and reviewed the coded list together. Topics associated with gender and age were identified and labeled in the transcript margins [36]. The coded data were examined to identify variations or contradictions in the answers. This analysis of the data led to format categories based on similarities, themes, or patterns of habits or norms. Further discussion between the authors and the research assistant enabled us to examine and produce subthemes from the responses. The three experts gave individual accounts that related to the core themes, which lends some credibility to the findings. The team resolved the minimal differences between coders and came to a consensus; the inter-rater reliability was = 0.95. For the results section, the most descriptive quotes for each question were selected, capturing the essence of the participants’ answers. 

## 3. Results

The themes were analyzed by gender and age groups: 

### 3.1. Body Awareness and Body Image 

The participants were asked about their body awareness, satisfaction, and dissatisfaction about appearance. It was found that almost all women in the younger age group (20–30 years) loved their bodies: “I live with it in peace”, whereas the least favorite body parts were the knees, hands, breasts (too big/too small), bottom, and belly. About 66% of woman in the second age group (31–40 years) did not love their bodies and were dissatisfied with their appearance. The main reason for dissatisfaction was that they felt overweight; in particular, they were dissatisfied with their bottom and belly. 

Regarding men, most of the younger participants were satisfied with their bodies; their favorite parts were their legs, hair, face, and eyes, and their least favorite body issues were teeth that stuck out, a large belly, a long, prominent nose, and noticeable ribs. However, only half of the second age group of men reported being satisfied with their bodies and their appearance. Their favorite body parts included the head, face, hair, and their upper-body muscles, whereas some of them were not satisfied with their belly and legs.

All men ages 20–30 reported that they did not have special eating habits. For example, “There is no strict adherence to healthy food; on the contrary—I eat everything, including fatty food and even at night”. Only two adults noted that they had poor eating habits: “I eat far more than I need, at bad hours, unhealthy food”. Another one reported he had changed his eating habits because of a medical condition (“I had cancer”). 

Seventy-five percent of the men in the second age group (ages 31 and over) indicated they had special eating habits, such as, “in the morning only cheeses and vegetables after the walk, no lunch and no fat food, only vegetables with chicken or fish, and in the evening, fruit or a cup of coffee”. Only one noted he had no eating habits: “Here and there I eat occasionally—a lot of snacks because of the patience needed to sit down and eat”.

Contrary to men, the younger women (ages 20–30) mentioned and detailed their eating habits and food that focused on “healthy eating”. Even if some women replied that they had “no eating habits”, they all noted they “tried to eat healthy”. Some of them stated that they ate healthy foods such as fruits and vegetables, and some clearly noted eating habits such as “six small meals a day, eating only when starving, focusing on vegetables and fish, lower-fat meat with nutritional supplements, in the morning coffee and an energy snack, a regular lunch, and in the evening something small”. Only one noted that having “bad eating habits—such as eating the remains of her children’s food”. Most of the women in the older age group reported they did not have special eating habits, and one woman said she did not eat meat.

The eating habits were an important topic in the participants’ answers. In general, most of the women said that their family emphasized healthy foods and healthy eating habits. For example, “my mother always told me to listen to my body”, and “both of my supervisors are on Weight Watchers so they maintain a healthy diet, and therefore we eat health food”. 

In addition, the participants were asked regarding sport activities. Half of the younger men reported that sports activities as a lifestyle for them such as football or attending the gym center. They noted that any sports activity might help “the body feel better, as well as its emotional affect. The men who were not engaged in any sport had some excuses such as past injuries. Most men ages 31–40 indicated they did not exercise on a regular basis, but they considered sports to be an important issue. They described their sports activities as going to the gym, surfing, biking, and playing handball, but they stopped for health reasons. The participants who exercised described the activity as “a great pleasure”. Some of them viewed sports as an opportunity to maintain a healthy body, “balance my cholesterol, lower my blood pressure, and maintains muscle flexibility”. Others wanted to “burn calories” and considered any sport as social entertainment while they were active. Within this age group, we noticed that this group also considers practicing sports to be a social activity. 

Women in the younger age group reported being mostly active in sports, had strict eating habits and tried to eat healthy, compared to men in their age group. Women in both age groups were more conscientious about eating healthy foods than the men were, whereas men in the older age group reported healthier eating habits than the younger men. Women appear to make a greater effort to form healthy food habits over the course of their lives, whereas men seem to change their eating habits as they get older. Half of the women ages 31–40 reported being occasionally engaged in doing sports activities. Some of the women had excuses for not regularly practicing a sport. One said that she stopped after getting pregnant; another stopped for medical reasons: “I had a surgery”. No significant differences were found between genders in engagement in sports activities. 

### 3.2. Family Impact

By exploring the perception the participants’ families, it was found that the family is the most powerful and important values and norms about the body and appearance. Following the interviews, it appears that most of the women ages 20–30 discussed their appearance or their bodies with their family members. They indicated they sought to hear the truth rather than “what you think you want to hear”. The women described their conversations with their mothers as significant when talking about their bodies and appearance. Participants also reported having received feedback and comments from their families on the participants’ need to lose weight. Although no homogeneity was found in the women’s responses, interesting to note that all the interviewees said their family members commented on their body appearance in one way or another. Some interviewees said the family feedback affected them, whereas others said it did not. However, they always discussed their appearance, especially with their mothers. One woman (age 24) said, “If they have compliments, they can say it, if something is bad—then they will keep it to themselves”. Another woman (25) said, “They know that I do not like this talk about my appearance, so they try not to talk about it” (age 24)”. Yet another (age 23) said, “It was a time when I was fatter and I had to lose weight. The comments were negative but I agreed with them. That was one of the things that helped me go on a diet”. Finally, one said, “My family usually was judgmental regarding my body and appearance. They always expressed positive and negative judgment”. In general, for all of the women, positive feedback from the family members was associated with looking slim or skinny, and negative feedback was associated with looking fat.

Among women in the second age group (31 to 40), large variation was present in the discourse on one’s body and appearance. Some women reported they did not talk at all with their families about their bodies or appearance; others talked about changes in their bodies because of births. Some did not perceive these conversations as meaningful, whereas others did. Most women described the negative affect of conversations with their parents. These negative comments were mostly about weight gain or unsuitable clothing, such as, “my parents’ remarks make me hate my body”. A few women complained that their fathers were “usually telling me and my sister that we are fat, and always told me we are not looking good enough”, or being confused by contradicting reactions to her appearance: “My parents said I was not pretty enough, but I got good feedback from my friends”. 

Some women remembered positive comments from their families, such as “you look good”. Other survey respondents remembered, “there were mostly positive comments and a lot of support” (age 33) and “no criticism, only sympathy; my parents knew that negative words make me feel sad”. One woman (age 32) remembered receiving exceptional support from her parents during adolescence: “My mother was very supportive and we talked about my body appearance a lot. They did not try to prevent me from doing surgery; on the contrary, they understood that it was important to me”. Women in this age group said that they mostly talked about appearance and body image with their children and a little with their extended family, “I talk about my body all the time; it’s very important to me. It is important for me to let my children be proud of themselves and to love the way they are and their bodies”.

When asked about family comments regarding their bodies and appearance, the young men (ages 20–30) noted that “the discussion was not important” or “it was not significant”. Family discussion focused either the need to be thin or on being too thin: “the family thought it was necessary to lose weight” or “I am constantly reminded about thinness, so I do not comment on anything and it does not bother me”. In general, the issue of family remarks seemed to be less important for the men than for the women. The group of older men indicated a varied discourse—both positive and negative—with their families about body appearance and performance. For example, one man (age 37) said, “My family says I look good”, whereas another (age 34) said a family member had commented, “Your belly grew; take care of yourself”.

In summary, family appears to have a significant influence on body perception, body image, and appearance among men and women in both age groups. The effect is complex and is expressed in a variety of responses and implications for both positive and negative performance and perception today.

### 3.3. Friends and Peers Impact

In this study, the interviewees discussed to the question regarding the impact of their friends, peers, or colleagues on their body perception. Women ages 20–30 indicated looking good was important to them. Some of them received positive responses, mainly comments regarding weight loss. One woman (age 24) said, “Friends express their opinion all the time in a positive way in the context of the body, in terms of overall appearance, clothing, and hair. As far as I was concerned, it was very flattering and confidence building”. Another woman (age 25) said, “My friends only comment if there is something positive”. Meanwhile, many women described negative comments. For example, one respondent (age 23) was told, “It would be nice to be less fat” and said “A colleague told me that sometimes I eat too much. Sometimes I agreed, especially when I was really fat, and sometimes I thought it was rude”. From participants’ disclosures, it appears that school peers’ comments might affect a person entire life. One woman (age 30) recalled, “In high school, boys used to call me ’fat girl’ for a long time. I pretended that I did not take it seriously, but I was sad”. 

Many women in the older age group reported receiving negative reactions to their appearance and said that the subject of thinness came up often. For example, “In elementary school, I was quite fat; friends were constantly talking about weight loss and diet” (age 33) and “negative judgments hurt and are insulting; this led me to have a negative perception of my body”. Today, looking good is important for most of them: “I compare myself with friends but I do not compete. I’m very careful about dress and it’s important to me what my friends say” (age 40). Among women in both age groups, comments regarding losing weight seem to have the most significant impact.

Like the women, men admitted that it was important for them to look good and getting reactions from their friends. However, in general, men seem to be less insulted or less hurt when talking about body weight. Men (ages 31–40) reported receiving fewer responses to their appearance today than they once did: “During the school period, I was at the beach a lot and I used to practice a lot, and my friends loved the muscles in my body” (age 34). Those that recalled recent responses reported they were often negative: “My friends express judgment on my belly. They are joking by asking, ‘What month of pregnancy are you?’ It is a little insulting, but they are right. They make comments about me being overweight, because they knew me a long time ago and see that I’m fat now” (age 33). 

In general, men in both age groups discuss weight and body appearance with their friends, but the discussion seems to be less significant than among the women. All men felt it is important to look good, but for the older adults, looking younger than their actual age was also important.

### 3.4. Media Impact

The media effect can be viewed as the external system that jointly influences the individual’s social, cultural, ideological, and belief systems. When asking about models in the media, the younger women admire female models. They love them because they are beautiful, and they love what they represent (age 28): “I admire an actress named Mila Kunis, who is married to Ashton Kutcher, because of her beauty”. Few women referred to male models, although one commented, “Mark is a Hawaiian player, looks good and is muscular; I tried to learn Capoeira because of him” (woman age 30).

Women ages 31–40 referred to role models who represent elegance, power, and/or wealth: “I liked Miss Arison (a rich and a powerful Israeli bank woman). There are many things to imitate: her conduct, the fact that she fulfills herself, and she looks good” (woman, age 39). For this age group of women, appearance does not appear to be the main characteristic they would like to imitate. 

Men in the younger age group reported that football players were their role models: “I really admire him because he was a good football player” (man, age 25). The interviewees also added that they liked the players “because of their abilities”. One of them (man, age 30) presented a complex picture, as he admired the beauty of artists, their character, and appearance”. “I have two models. I imitate their self-confidence, as I put my hands in my pockets and talk like them”.

For men in the second age group, friends and family members became their role models. One man (man, age 34) said, “My son is a role model of how I would like to see myself. He is just as I was young”. Others said, “I had a friend who was a singer; I admired him” (man, age 37) and “My grandfather was my model: he was tall, dressed well, and always said to raise the head, to walk straight ahead”. 

Regarding the media effects, from our interviews, role models can be famous artists or people recognized from the past. Imitation mainly existed among the younger age group. 

### 3.5. Impact of Body Image and Appearance on Workers’ Careers

Within this study, most of the participants addressed the importance of one’s look and appearance at work, primarily in job interviews. However, gender differences revealed that men are less offended when talking about weight. Women emphasized the mixture of good or nice appearances with the benefit of succeeding at work. “My good appearance helped. Being good looking opened opportunities for me” (woman, age 32). “My good appearance helped a lot; for example, in work interviews, people like to see good-looking employee, and the boss might give me a higher position” (woman, age 40). Some of the respondents reflected on the negative aspect of body appearance and its effects on jobs or promotions: “A friend that dresses good was promoted” (man, age 31). “Yes, I think that appearance always helps the way people are treated and it affects whether you get the job or not. Weighing more will make other people look at me different” (man, age 33). “A bookkeeper said that I was fat and not appreciated; although in practice, I was a professional” (man, age 37). In addition, participants reported on close relationships between body image and appearance though performance at work. For example, “In my work interview, how I look is one of the aspects that determines whether I get the job or not” (woman, age 22); and “The way my body looks affect how I feel” (woman, age 31). 

## 4. Discussion

Our results revealed that participants were concerned about their general body appearance, including their weight, across age groups and gender. Although many people were unsatisfied with their appearance and/or their weight, they failed to persist with sports and/or dieting. These results are in line with Bibiloni et al.’s [34] showing that 75% of the Mediterranean adult population reported body-image dissatisfaction, but almost half of them reported not being worried about their body weight. Females were more likely to be concerned about their body weight than males. This study assessed body image among people in the Israeli’ adult population. According to the media, the ideal body appearance is tall and thin, which typically does not reflect the average woman or man. Our results support the notion that women are more likely to be concerned about their body appearance than men. This result reflects the results of the meta-analysis by Gentile et al. [6] in which older participants were less satisfied with their body image, and men reported on higher and positive physical appearance than female. In addition, our findings revealed that the younger group, both men and women, were more satisfied with their body appearance than the older groups of both genders. This result may reflect the concerns of individuals as they get older, as they are not happy with their body changes.

In addition, young women reported having heathy eating habits, whereas the older group of women did not. Although it is not a causal statistical study, our research shows that the relationship between no healthy eating habits, increased weight, and being only partly active in sports is apparent. The implications of these findings could be applicable across country, age, and gender: the triangular connection between age, eating habits, and sports must begin at an early age.

Implementing specific learning programs from kindergarten through adulthood regarding heathy eating norms and outdoor activities might be worthwhile. In particular, the findings revealed the significance of the parents’ comments on one’s perceived body appearance, especially among women. The present findings are consistent with previous findings regarding Israeli social interactions in the family [10,11]. The family serves as a central social agent, which affects the individual. Therefore, prevention and intervention programs aimed at supporting parents and their children through learning heathy eating habits and the importance of physical exercise in order to maintain a positive body image as one gets older could be valuable.

The present study examined the relationship between social-environmental influences and body-image perception. Family habits and parents’ spoken observations (positive and negative) about their children’s bodies had an ongoing and significant influence on the participants’ body appearance and body-image perceptions among both genders in all both groups. Even among men, in both age groups, thinness and impressive body presentation took up significant social space; all of the men felt that looking good, appearing attractive, and wearing fashionable clothing was important, and the older group felt that looking younger than their chronological age was especially important. However, the subject of body appearance was less emphasized among men than among women. 

Frisén and Holmqvist [37] noted that during adolescence, conversations with family members about appearance often focus not on the body itself but on more external and changeable aspects, such as clothes and hairstyle, and that girls generally receive more positive feedback about their appearance than boys. In the present study, a similar pattern was found among young-adult and adult women. Thus, in both age groups, more women received positive and negative comments about their body appearance from family and friends, and these comments were more significant to them compared to men. Results also revealed that for the younger group that young women appear to be more likely than men to admire people in the media and consider them role models. Respondents’ focus was on external beauty or physically attractiveness and muscles. The older age group of both genders appreciated the media personality’s personal characteristics or the model’s achievement and fulfilment, and even admired a friend or a family member as a role model for their own children. In other words, contrary to the younger age group, the older anticipants focused not only on the body appearance, but also on the added value of the role model as a person. 

At work, respondents of both genders maintained a dignified appearance and reported that body appearance had an effect on their success at work or in their careers. However, participants indicated that external appearance is insufficient for career success, and that bosses and co-workers also judge them for their professionalism and integrity. 

## 5. Conclusions

The present findings contribute to our knowledge regarding the relationship between social and psychological experience related to body perceptions, and highlight the vulnerability of both genders. The findings indicated that women tend to have lower body image than men, are more concerned with weight and dieting, and exercise less than men. This study might be the first attempt to examine body issues and the impact of the family, social environment, and media according to the individual’s gender and age. Although the study did not focus on the causality model, the findings expressed participants’ needs for specific help to increase awareness of self-perception, well-being, and body perception in order to reduce their negative experiences. 

The present study can be viewed as a pilot study. The findings present the voice of 30 students, representing a homogeneous socioeconomic and Israeli-Jewish culture; the participants belong to middle and upper class, Caucasian sample. Therefore, the results should be cautious about generalization of the qualitative findings to populations that differ in social class, religion, and ethnicity. Additional studies are needed to explore the experiences of adults after a training program, and to verify the program’s effects on body image. These future studies need to undertake comprehensive, longitudinal, in-depth mixed-methods, including questionnaires and interviews, including a variety of adults with different backgrounds, which might deepen our understanding of body self-perception. 

## Figures and Tables

**Table 1:** Participants’ background.

Participant	Marital Status	Education	Age
Women			
1.	unmarried	BA	22
2.	unmarried	BA	22
3.	unmarried	BA	22
4.	unmarried	BA	23
5.	unmarried	BA	23
6.	unmarried	BA	23
7.	married	BA	23
8.	married	BA	24
9.	married	BA	24
10.	married	BA	25
11.	married	BA	31
12.	married	MA	32
13.	unmarried	MA	31
14.	married	MA	40
15.	married	BA	33
16.	married	BA	32
Men			
1.	unmarried	BA	23
2.	unmarried	BA	23
3.	unmarried	BA	24
4.	unmarried	BA	25
5.	unmarried	BA	25
6.	unmarried	BA	25
7.	unmarried	BA	27
8.	unmarried	BA	27
9.	married	BA	31
10.	married	BA	31
11.	married	BA	33
12.	married	MA	34
13.	divorced	MA	37
14.	divorced	BA	34

## Appendix A

**Section I: Body awareness and body image**

1. How do you perceive your body?

2. What do you think about your body? Which parts of your body do you like most and which less?

3. Are you doing any sports? Do you follow any special eating habits?

**Section II: Impact of agents of socialization and on body image**

1. Did you ever speak of body and appearance in your family?

2. Did your family members express any judgments, thoughts, and feelings about your body and body appearance?

3. Did your friends, peers, and colleagues express any judgments, thoughts, and feelings about your body and body appearance?

4. Did or do you have a celebrity as a model (as in the media, sports, in fashion, films, etc.) that you admire for their appearance?

**Section III: Impact of body image and appearance on workers’ careers**

1. Do you think that your body image and appearance have had an influence in your working career? Did they help you? Do you think that your body image and appearance affect your job interviews?

2. Do you think that also your body image and appearance have affected your career promotion?

## References

[1] Cash T.F. (2004). Body image: Past, present and future. Body Image.

[2] Cash T.F., Cash T.F., Pruzinsky T. (2002). A ‘negative body image’: Evaluating epidemiological evidence. Body Image: A Handbook of Theory, Research, and Clinical Practice.

[3] Sondhaus E.L., Kurtz R.M., Strube M.J. (2001). Body attitude, gender and self-concept: A 30-year perspective. J. Psychol. Interdiscip. Appl..

[4] Grabe S., Ward J.S., Hyde L.M. (2008). The role of the media in body image concerns among women: A meta-analysis of experimental and correlational studies. Psychol. Bull..

[5] Cash T.F., Pruzinsky T., Cash T.F., Pruzinsky T. (2002). Future challenges for body image theory, research, and clinical practice. Body Image: A Handbook of Theory, Research, and Clinical Practice.

[6] Gentile B., Grabe S., Dolan-Pascoe B., Twenge J.M., Wells B.E., Maitino A. (2009). Gender differences in domain-specific self-esteem: A meta-analysis. Rev. Gen. Psychol..

[7] Tiggermann M. (2004). Body image across the adult lifespan: Stability and change. Body Image.

[8] Amianto F., Martini M., Spalatro A., Abbate D.F., Fassino S. (2017). Body Image Development within the Family: Attachment Dynamics and Parental Attitudes in Cross-Sectional and Longitudinal Studies. Acta Psychopathol..

[9] Muth J.L., Cash T.F. (1997). Body-image attitudes: What difference does gender make?. J. Appl. Soc. Psychol..

[10] Harel-Fisch Y., Zhou H., Yat Ming F. Family Structure, School Climate and Drinking Habit of Adolescent Boys in China: A Multi-Level Analysis. Proceedings of the WHO-Health Behavior in School-Aged Children Annual Meeting.

[11] Katz B. (2013). Eating behavior and risks of eating disorders among adolescents in Israel. Mifgash J. Soc.-Educ. Work.

[12] Miller M.N., Pumariega A.J. (2001). Culture and eating disorders: A historical and cross-cultural review. Psychiatry.

[13] Swami V. (2015). Cultural influences on body-size ideals: Unpacking the influence on Westernizations and modernization. Eur. Psychol..

[14] Becker C.B., Diedrichs P.C., Jankowski G. (2013). I’m not just fat, I’m old: Has the study of body image talk overlooked ‘old talk?. ’ J. Eat. Disord..

[15] Jankowski G.S., Diedrichs P.C., Williamson H., Christopher G., Harcourt D. (2014). Looking age-appropriate while growing old gracefully: A qualitative study of ageing and body image among older adults. J. Health Psychol..

[16] Bergman Y.S., Bodner E., Cohen-Fridel S. (2012). Different dimensions of ageist attitudes among men and women: A multigenerational perspective. Int. Psychogeriatr..

[17] Cheng H.L., Mallinckrodt B. (2009). Parental bonds, anxious attachment, media internalization, and body image dissatisfaction: Exploring a mediation model. J. Couns. Psychol..

[18] Hardit S.K., Hannum J.W. (2012). Attachment, the tripartite model, and the development of body dissatisfaction. Body Image.

[19] Arroyo A., Segrin C., Andersen K.K. (2017). Intergenerational transmission of disordered eating: Direct and indirect maternal communication among grandmothers, mothers, and daughters. Body Image.

[20] Kluck A.S. (2010). Family influence on disordered eating: The role of body image dissatisfaction. Body Image.

[21] Paxton S.J., Eisenberg M.E., Neumark-Sztainer D. (2006). Prospective predictors of body dissatisfaction in adolescent women and men: A five-year longitudinal study. Dev. Psychol..

[22] Tatangelo G., Ricciardelli L. (2017). Children’s body image and social comparisons with peers and the media. J. Health Psychol..

[23] Sheldon P. (2013). Testing parental and peer communication influence on young adults’ body satisfaction. South. Commun. J..

[24] Jhaveri S., Patki S.M. (2016). Locus of control, peer influence on dieting, media exposure and body image satisfaction in young adults. Indian J. Health Wellbeing.

[25] Ridolfi D.R., Myers T.A., Crowther J.H., Ciesla J.A. (2011). Do appearance focused cognitive distortions moderate the relationship between social comparisons to peers and media images and body image disturbance. Sex Roles.

[26] Ferguson C.J. (2013). In the eye of the beholder: Thin-ideal media effects some, but not most, viewers in a meta-analytic review of body dissatisfaction in men and women. Psychol. Pop. Media Cult..

[27] Frederick D.A., Peplau L.A., Lever J. (2006). The swimsuit issue: Correlates of body image in a sample of 52,677 heterosexual adults. Body Image.

[28] Myers T.A., Crowther J.H. (2009). Social comparison as a predictor of body dissatisfaction: A meta-analytic review. J. Abnorm. Psychol..

[29] Fardouly J., Diedrichs P.C., Vartnian L.R., Halliwell E. (2015). Social comparisons on social media: The impact of Facebook on young women’s body image concerns and mood. Body Image.

[30] Fardouly J., Vartanian L.R. (2015). Negative comparisons about one’s appearance mediate the relationship between Facebook usage and body image concerns. Body Image.

[31] Barlett C.P., Harris R.J. (2008). The impact of body emphasizing video games on body image concerns in men and women. Sex Roles.

[32] Bell B.T., Dittmar H. (2011). Does media type matter? The role of identification in adolescent women’ media consumption and the impact of different thin-ideal media on body image. Sex Roles.

[33] Holmstrom A.J. (2004). The effects of the media on body-image: A meta-analysis. J. Broadcast. Electron. Media.

[34] Bibiloni M., Coll J.L., Pich J., Pons A., Tur J.A. (2017). Body image satisfaction and weight concerns among a Mediterranean adult population. BMC Public Health.

[35] Willig C. (2001). Introducing Qualitative Research in Psychology.

[36] Ritchie J., Spencer L., O’Connor W., Ritchie J., Lewis J. (2003). Carrying out Qualitative Analyses. Qualitative Research Practice: A Guide for Social Science Students.

[37] Frisén A., Holmqvist K. (2010). What characterizes early adolescents with a positive body image? A qualitative investigation of Swedish girls and boys. Body Image.

